# Iodine intake assessment in the staff of a Porto region university (Portugal): the iMC Salt trial

**DOI:** 10.1007/s00394-023-03149-1

**Published:** 2023-04-20

**Authors:** Ana Machado, Carla Gonçalves, Pedro Moreira, Olívia Pinho, Patrícia Padrão, Tânia Silva-Santos, Micaela Rodrigues, Pedro Norton, Adriano A. Bordalo

**Affiliations:** 1 ICBAS—Instituto de Ciências Biomédicas Abel Salazar, University of Porto, Rua Jorge Viterbo Ferreira 228, 4050-313 Porto, Portugal; 2 CIIMAR – Interdisciplinary Centre of Marine and Environmental Research, University of Porto, Novo Edifício do Terminal de Cruzeiros do Porto de Leixões, Avenida General Norton de Matos, S/N, 4450-208 Matosinhos, Portugal; 3 EPIUnit—Instituto de Saúde Pública, Universidade do Porto, 4050-091 Porto, Portugal; 4 ITR – Laboratory for Integrative and Translational Research in Population Health, Rua das Taipas 135, 4050-600 Porto, Portugal; 5 CITAB - Centre for the Research and Technology of Agro-Environmental and Biological Sciences, 5001-801 Vila Real, Portugal; 6 Faculty of Nutrition and Food Sciences, University of Porto, 4099-002 Porto, Portugal; 7 LAQV/REQUIMTE - Laboratório de Bromatologia e Hidrologia, Departamento de Ciências Químicas, Universidade do Porto, Porto, Portugal; 8 Departamento de Saúde Ocupacional, Centro Hospitalar Universitário São João, Porto, Portugal

**Keywords:** 24-h urinary iodine excretion, Iodine intake, 24-h dietary recall, Salt intake, Adult population, Public health

## Abstract

**Purpose:**

Iodine deficiency disorder (IDD) is an ongoing worldwide recognized problem with over two billion individuals having insufficient iodine intake. School-aged children and pregnant women are often target groups for epidemiological studies, but there is a lack of knowledge on the general adult population. The aim of this study was to assess the iodine status among a Portuguese public university staff as a proxy for the adult working population.

**Methods:**

The population study covered 103 adults within the iMC Salt randomized clinical trial, aged 24–69 years. Urinary iodine concentration was measured spectrophotometrically using the Sandell–Kolthoff reaction. Iodine food intake was assessed using a 24-h dietary recall. The contribution of discretionary salt to the iodine daily intake was assessed through 24-h urinary sodium excretion (UIE) and potentiometric iodine determination of household salt.

**Results:**

The mean urine volume in 24 h was 1.5 L. The median daily iodine intake estimated from 24-h UIE was 113 µg/day, being lower among women (*p* < 0.05). Only 22% of participants showed iodine intake above the WHO-recommended cutoff (150 µg/day). The median daily iodine intake estimated from the 24-h dietary recall was 58 µg/day (51 and 68 µg/day in women and men, respectively). Dairy, including yoghurt and milk products, were the primary dietary iodine source (55%). Iodine intake estimated from 24-h UIE and 24-h dietary recall was moderately correlated (Spearman rank correlation coefficient *r* = 0.34, *p* < 0.05). The average iodine concentration in household salt was 14 mg I/kg, with 45% of the samples below the minimum threshold preconized by WHO (15 mg I/kg). The contribution of discretionary salt to the daily iodine intake was around 38%.

**Conclusion:**

This study contributes new knowledge about iodine status in Portuguese working adults. The results revealed moderate iodine deficiency, particularly in women. Public health strategies and monitoring programs are needed to ensure iodine adequacy in all population groups.

## Introduction

Iodine is an essential micronutrient, primarily obtained from the diet, indispensable to thyroid hormone production and key for the metabolism regulation of mammals [1]. Both, low and high iodine intake can lead to thyroid dysfunction [2]. In children, the adverse effects of its deficiency include intellectual impairments and growth retardation, while in adults it has been linked to goiter, increased risk of thyroid cancer and clinical manifestations of hypothyroidism, such as reduced metabolic rate, cold intolerance, weight gain, puffy face, edema, hoarse voice and mental sluggishness. Excessive iodine intake, on the other hand, can also induce thyroid dysfunction resulting in both hyper- and hypothyroidism, increased risk of cancer and thyroid autoimmunity [3].

Iodine deficiency disorder (IDD) is still an ongoing worldwide recognized problem, with over two billion individuals having inadequate iodine intake [2]. Although often seen as a problem in developing countries, IDD has re-emerged in the industrialized world, including regions previously iodine sufficient [3]. Despite the global effort to tackle the problem, recently a high number of European countries (44%) have been reported as iodine deficient, and updated data regarding the status of iodine in different populations remains insufficient [4, 5].

In Portugal, studies reported that the median urinary iodine concentration (UIC) is generally adequate in school-aged children [5–7]. Nevertheless, iodine inadequacy was found in pregnant women more than a decade ago [8], and although a recent supplementation approach was implemented, iodine adequacy has not yet been achieved [5, 9]. Unfortunately, the current iodine status of the broad Portuguese population is not available.

Since about 90% of ingested iodine is excreted in the urine, the median UIC is recognized as a good biomarker of short-term iodine status in groups [10]. According to the World Health Organization (WHO) criteria, iodine nutrition status in school-age children and non-pregnant adults is insufficient when median UIC is below 100 μg/L, adequate when between 100 and 199 μg/L (and < 20% of the population with UIC ≤ 50 μg/L), more than adequate when between 200 and 300 μg/L, and excessive when higher than 300 μg/L [1].

The main sources of iodine in the human diet are marine foodstuffs—fish, shellfish, algae and sea salt. Water, milk, vegetables and processed foods, such as bread and margarine, are also potential iodine sources [2]. The WHO recommends a daily iodine intake of 90 μg for children 0–5 years, 120 μg for children 6–12 years, 150 μg for adolescents and adults, and 250 μg for pregnant and lactating women [10].

Universal salt iodization is preconized by the WHO, the United Nations Children's Fund (UNICEF) and the Iodine Global Network (IGN) as a safe, cost-effective and sustainable way to tackle IDD [1]. Worldwide, 88.7% of the population uses iodized salt, even though there is no data concerning the current situation in most European countries [11]. Portugal lacks a mandatory salt iodization policy, with iodization being optional for household and industry salt since 1996 [12]. Due to the IDD challenges and the lack of a regulatory framework, the use of fortified salt in Portuguese school canteens has been recommended since 2013 [6, 13]. Presently, additional concern arose in the vein of the WHO recommendation to reduce salt intake to 5 g of per day, to prevent hypertension and cardiovascular disease [14], and the potential impact of this policy on the iodine nutritional status.

Therefore, the aim of this study was to evaluate the iodine nutritional status of the public University of Porto staff, as a proxy for the Portuguese adult working population. Additionally, it was intended to demonstrate the potential of occupational health appointments in the education and monitoring of the iodine subject.

## Materials and methods

### Population and study design

The participants of the study were recruited to the iMC Salt randomized controlled trial, designed to decrease salt intake inadequacy. The iMC Salt study was designed to assess the effectiveness of interventions to reduce salt consumption among consumers. The intervention consisted of using the Salt Control H equipment by the participants at home to control the salt quantity used for cooking all meals during the intervention period. Salt Control H is a dispenser that offers doses of salt according to the number of persons and the age (child or adult) of the consumers. The study was an 8-week randomized controlled trial, with follow-up to week 35. Participants were recruited among the staff of the university through the annual mandatory occupational health appointments. The exclusion criteria were pregnancy, hypotensive disorder, renal infection, kidney disease, urinary incontinence, acute coronary syndrome, severe liver disease or heart failure, member of the faculty that promotes the study (i.e., Faculty of Nutrition and Food Sciences), and not using salt for cooking. The primary outcome of this study was the difference in 24-h urinary sodium (as a proxy of salt intake) between the intervention and the control group from the baseline to the end of the intervention (week 8). A detailed description of the methods of the iMC Salt study has been published elsewhere [15, 16].

The iodine nutritional status was assessed for the baseline participants group, after validation of the collected 24-h urine samples, and additional exclusion of participants with previously diagnosed thyroid disease. A sample size of *n* = 131 in the iMC Salt trial was initially estimated to provide 80% power and *p* < 0.05 (two sided) to reject the null hypothesis [16]. However, due to restrictions imposed by the COVID-19 pandemic only 103 participants were enrolled in the iodine assessment study. After giving informed, written consent, anthropometric measurements (weight, height) were collected. Additionally, participants were asked to answer a sociodemographic questionnaire based on the WHO STEPS questionnaire [17], to provide information about age, sex, education, smoking, and other lifestyle characteristics.

### 24-h urine collection

As previously reported [16], each participant was instructed (oral and written) to collect urine during a 24-h period. Briefly, the individual should void the bladder and discard the first morning urine collection, and then collect all urine during the following 24-h period, including the morning void of the day after. Any missed voids or irregularities should be recorded. After returning the containers, the 24-h urine collection was well stirred and the volume recorded. Urine aliquots were then extracted and stored at − 80 °C until analysis.

### Estimation of dietary and discretionary salt iodine content

Food intake was assessed using a 24-h dietary recall. Participants were asked to recall all foods and beverages consumed the day before (time of urine collection) and to estimate the portion size with the help of a picture book. The conversion of food intake into iodine was performed using the Food Processor Plus software (ESHA Research, Inc., Salem, OR USA) adapted for the Portuguese population [18]. The nutrient content of local food was taken from standard nutrient tables [18], whereas the content of commercial food (e.g., pizza and ready-to-eat-food) was derived from labeled ingredients and nutrients.

The food were categorized into 15 major food groups [19]: (1) cereals, derivatives and tubers (pasta, rice and other cereal-based products, potatoes and other tubers, bread and toasts, infant cereals and breakfast cereals); (2) meat and eggs (white and red meats, ham, bacon, sausages and all processed meats, eggs); (3) fresh, dried and canned fish (fresh fish and roe, cod fish and smoked salmon and canned fish); (4) crustaceans, mollusks and others (octopus, squid, shrimp, clams, mussels, oysters, including preserves); (5) processed fish (fish nuggets, surimi, fish pastes, pâtés and other processed fishes); (6) dairy products (milks, yogurts and cheeses); (7) soups (vegetable soups, meat and fish soups and chicken soups); (8) sweets, cakes and cookies (sugars, chocolates and chocolate snacks; ice cream, sweet desserts; cakes, pies and confectionery; biscuits); (9) snacks and pizzas (bread snacks, fried potatoes, salted popcorn, salted snacks, fried snacks and pizzas); (10) fruits, vegetables and pulses (fresh and processed vegetables, nuts and seeds, fresh, canned and dried fruit, fresh and dried pulses); (11) non-alcoholic beverages (water, tea and infusions, coffee, juices and soft drinks and other non-alcoholic beverages); (12) other foods (herbs and spices, sauces and condiments, broths and instant soups); (13) oils and fats (vegetable oils, olive oil, margarine, butter and other fats); (14) alcoholic beverages and (15) meat and milk substitutes. For each subject, all food described in the 24-h dietary recalls were allocated into those 15 major food groups. The quotient between the sum of iodine values for each food groups and the estimated total value of the iodine intake was calculated. The proportion across participants of each food group to total iodine intake was obtained.

In the context of the iMC Salt study, the total sodium consumption was assessed through 24-h urinary excretion and a 24-h dietary recall [15]. Added sodium was calculated by the difference between the sum of sodium values of foods in each group and the urinary sodium excretion value. After the calculations, the sodium was converted to salt. Each participant assigned to the iMC Salt randomized controlled trial intervention group was asked for a household salt sample. The iodine content of household salt samples was measured in triplicate, by potentiometry using a combined iodide electrode (HI 4111, Hanna Instruments, USA), following Lobato et al. [20].

### Determination of urinary iodine and creatinine concentration

Urinary iodine concentration was assessed spectrophotometrically, using a modification of the Sandell–Kolthoff reaction, with an initial ammonium persulfate digestion [21]. A standard calibration curve was obtained for each batch of digested samples. The laboratory team participates in the ongoing international CDC-Atlanta inter-calibration program Ensuring the Quality of Urinary Iodine Procedures (EQUIP), for the determination of iodine in human urine samples [22]. The results were expressed as the median and interquartile range for the descriptive statistics for easy comparison with reference values and the iodine status reported in other studies. The median UIC of the participants was compared with the iodine status criteria preconized by the WHO, UNICEF, and IGN, for adults [1], assuming that the 24-h UIC value from each participant was the mean of several spot urine samples. Daily urinary iodine excretion was calculated by multiplying the UIC by the 24-h urine volume and expressed as micrograms per 24 h. Daily iodine intake was estimated in micrograms per day assuming 92% of bioavailability, and 90% of the ingested iodine excreted in urine [4], using the formula UIE/0.83. The prevalence of inadequate iodine intake was estimated according to the WHO [1] and the European Food Safety Authority (EFSA)-recommended intake of 150 µg/day and tolerable upper intake of 600 µg/day iodine values for adults [23].

Urinary creatinine, measured by the kinetic Jaffé reaction [24] with direct colorimetric detection, was used as an indicator to assess the adequacy of the 24-h urine collection.

The researcher who performed the urinary measurements was not aware of which group within the iMC Salt trial the participant was allocated to.

### Ethics

Ethical approval for the study was obtained from the Ethics Committee of the Centro Hospitalar Universitário São João, trial registration number NCT03974477. All participants signed an informed consent statement before the start of the study.

### Statistical analysis

Analysis of the data was performed using STATISTICA software (V.7.0 Stat Soft Inc., Tulsa, OK, USA). Descriptive statistics are presented as mean with standard deviation (SD) and medians, with 25th and 75th percentiles.

Spearman’s correlation coefficient was used for the comparison of iodine intake estimated from 24-h urine and 24-h dietary recall. Bland–Altman plots were constructed to evaluate the agreement between the two iodine intake measurement techniques applied.

Mann–Whitney *U* test was used to test differences in UIC within the different dietary practices.

One-way analysis of variance with the Kruskal–Wallis test and Dunn’s multiple comparison post-test was used to explore differences between groups. The level of significance was defined as a *p* value < 0.05.

## Results

### Characteristics of the iMC Salt participants

The study enrolled 103 participants, most (61%) living near coastal areas, evenly distributed by sex (49.5% women), with a mean age of 47 years (Table 1). The average body mass index was 26 ± 3.9 kg/m^2^, with most women showing a normal weight, whereas the large majority of men evidenced overweight or even obesity (*p* < 0.05). Most participants had a university degree (86%) and 12% reported smoking at the time of recruitment. The weekly alcohol consumption averaged 0.6 L, with a higher consumption volume in men. Only 10% of the participants reported the use of unspecified nutritional supplements.

**Table 1:** iMC Salt baseline population characteristics (*n* = 103)

	All	Women	Men	*p* value
Sex, *n*	103	51	52	
Age, years	47 (10.6)	45 (9.9)	49 (11)	0.121
Body mass index (BMI), kg/m^2^	26 (3.9)	25 (3.9)	27 (3.6)	< 0.001
Underweight, %	14 (2)	4 (2)	0 (0)	
Normal weight, %	37 (38)	53 (27)	22 (11)	
Overweight, %	47 (48)	33 (17)	61 (31)	
Obesity, %	15 (15)	10 (5)	20 (10)	
Education level				0.483
High school or less, %	14 (14)	18 (9)	10 (5)	
University, %	86 (89)	82 (42)	90 (47)	
Smoking habits				0.750
Non-smoker, %	65 (67)	71 (36)	60 (31)	
Smoker, %	12 (12)	10 (5)	13 (7)	
Former smoker, %	23 (24)	20 (10)	27 (14)	
Weekly alcohol consumption, L	0.6	0.4	0.8	0.522
Abstainers, %	10 (10)	12 (6)	8 (4)	
Up to 1 L^a^, %	76 (78)	80 (41)	71 (37)	
More than 1 L, %	15 (15)	8 (4)	21 (11)	
Supplementation				0.987
No, %	10 (10)	10 (5)	10 (5)	
Yes, %	90 (93)	90 (46)	90 (47)	
Residence area				0.279
Coastal, %	61 (63)	55 (28)	67 (35)	
Inland, %	39 (40)	45 (23)	33 (17)	

Values are mean (standard deviations) or percentages (numbers)

Coastal—Espinho, Matosinhos, Ovar, Porto, Póvoa do Varzim, Vila Nova de Gaia

Inland—Albergaria-a-Velha, Amarante, Braga, Famalicão, Gondomar, Guimarães, Maia, Paços de Ferreira, Paredes

^a^1 L per week corresponding to up to a glass per day (0.14 L)

### Iodine excretion and estimated iodine intake

The median 24-h urine volume for the study population was 1.5 L (IQR 1.0, 1.8), with no statistical difference between sex, age, or BMI (*p* > 0.05) (Table 2).

**Table 2:** iMC Salt baseline participants’ mean, median, and interquartile ranges of 24-h urine volume, 24-h urine iodine concentration (24-h UIC), 24-h iodine excretion (24-h UIE), and 24-h iodine intake, by sex

	All (*n* = 103)	Women (*n* = 51)	Men (*n* = 52)	*p* value
24-h urine volume, L				0.136
Mean (SD)	1.5 (0.6)	1.4 (0.6)	1.6 (0.6)	
Median (25th, 75th)^a^	1.5 (1.0, 1.8)	1.31 (1.0, 1.8)	1.6 (1.2, 1.9)	
24-h UIC, µg/L				0.765
Mean (SD)	72 (36)	71 (37.81)	73 (34)	
Median (25th, 75th)^a^	66 (42, 91)	74 (40, 91)	65 (46, 90)	
24-h UIE, µg/day^b^				0.022
Mean (SD)	100 (46)	90 (43)	110 (48)	
Median (25th, 75th)^a^	94 (68, 120)	85 (65, 113)	103 (77, 142)	
24-h iodine intake, µg/day^c^				0.022
Mean (SD)	121 (56)	109 (51)	133 (58)	
Median (25th, 75th)^a^	113 (82, 145)	102 (78, 136)	124 (93, 171)	
% ≥ 150 µg/day (n)	22 (23)	14 (7)	31 (16)	

SD, standard deviation; UIC, 24-h urine iodine concentration; UIE, 24-h iodine excretion

^a^25th percentile and 75th percentile

^b^24-h iodine excretion (µg/day) = UIC (µg/L) × 24-h urine volume (L/day)

^c^24-h iodine intake, estimated from 24-h UIE divided by intake/excretion ratio of 0 83

The median 24-h UIC was 66 µg/L (IQR 42, 91), slightly lower in men (74 µg/L and 65 µg/L in women and men, respectively). To note that taking into account the statistical descriptor mean, the 24-h UIC was threshold higher in men (Table 2). Statistically significant higher 24-h UIC values were reported in the university educational level participants (*p* = 0.01). Only 17% of the participants had 24-h UIC values in the optimal range, with no statistical difference between sex. About 80% of the participants exhibited UIC values below 100 µg/L, among which 30% presented 24-h UIC values below 50 µg/L. In this study, 24-h UIC values above 200 µg/L were not observed (Table 3).

**Table 3:** Distribution of the iMC Salt baseline participants by WHO epidemiological criteria for assessing iodine nutrition status in a population, based on urine iodine concentration (UIC)

UIC (µg/L)	Iodine intake	Iodine nutrition status	All (*n* = 103)	Women (*n* = 51)	Men (*n* = 52)
< 20	Insufficient	Severe deficiency	2% (2)	4% (2)	–
20–49	Insufficient	Moderate deficiency	30% (31)	31% (16)	29% (15)
50–99	Insufficient	Mild deficiency	50% (52)	49% (25)	52% (27)
100–199	Adequate	Optimal	17% (18)	16% (8)	19% (10)
200–299	Above requirements	Slight risk	–	–	–
≥ 300	Excessive	Risk	–	–	–

Values are percentages (numbers)

The median 24-h population UIE was 94 µg/day (IQR 68, 120), statistically significantly higher in men (*p* = 0.02). The median daily iodine intake estimated from UIE was 113 µg/day (IQR 82, 145), with statistically significant lower values in women (*p* = 0.02). Only 22% of participants (14% of the women, 31% of the men) had iodine intake above the WHO and EFSA recommended cutoff value for adequate iodine intake (150 µg/day).

### Estimated iodine intake from the 24-h dietary recall

The estimated daily iodine intake of the participants who fully completed the 24-h dietary recall and simultaneously had a valid urine sample (*n* = 92), ranged from 0.56 to 272 µg/day, with a median iodine intake of 58 µg/day (IQR 30, 97) (Table 4).

**Table 4:** iMC Salt baseline participants mean, median, and interquartile ranges of estimated iodine intake (µg/day) from the dietary recall, by sex

	All (*n* = 92)	Women (*n* = 48)	Men (*n* = 44)	*p* value
24-h DR iodine intake, µg/day				0.161
Mean (SD)	69 (55)	64 (59)	74 (50)	
Median (25th, 75th)^a^	58 (30, 97)	51 (26, 85)	68 (41, 99)	

24-h DR, 24-h dietary recall; SD, standard deviation

^a^25th percentile and 75th percentile

The 24-h dietary recall showed a slightly lower iodine intake in women, and as opposed to the iodine intake estimated from UIE, no statistical difference between sex was observed. Only 8.3% of women and 6.8% of men had iodine intake equal to or above the recommended daily intake (150 µg/day). No participants evidenced iodine intake above the tolerable recommended upper limit (UL < 600 µg/day).

### Relationship between the two methods for estimating iodine intake

The median iodine intake estimated from the 24-h dietary recall (58 µg/day) was significantly lower than the levels determined from the UIE (113 µg/day, *p* < 0.05). A significant moderate correlation between the iodine intake estimated from the two methods could be established (Spearman rank correlation coefficient *r* = 0.34, *p* < 0.05). The correlation was slightly higher when only the iodine intake of women was analyzed (*r* = 0.39, *p* < 0.05).

**Fig. 1:**
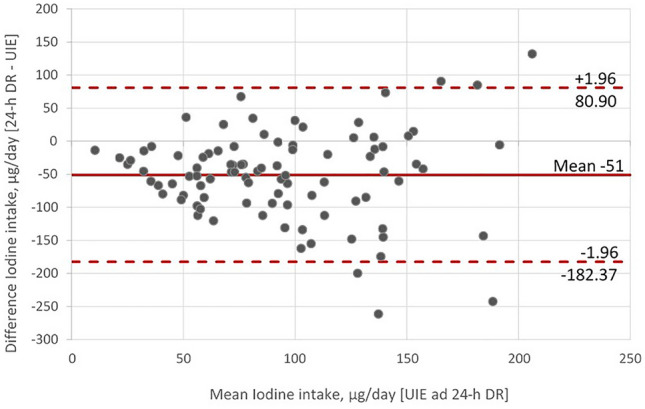
Bland–Altman plot, mean iodine intake estimated from 24-h urine iodine excretion (UIE) and the 24-h dietary recall (24-h DR) versus the difference between the methods (24-h DR—UIE). The red line indicates the mean difference in iodine intake estimates, whereas the red dashed line represents the limits of agreement from − 1.96 SD to + 1.96 SD

The agreement between different determination approaches was assessed through a Bland–Altman plot (Fig. 1). The analysis showed that the 24-h dietary recall underestimated the intake of iodine when compared with the UIE. Moreover, the differences between the methods increased with increasing intake. The mean differences were − 51 (SD 67) µg/day, slightly higher in men (− 59 µg/day, SD 67) than in women (− 43 µg/day, SD 67).

### Sources of iodine from the 24-h dietary recall

**Fig. 2:**
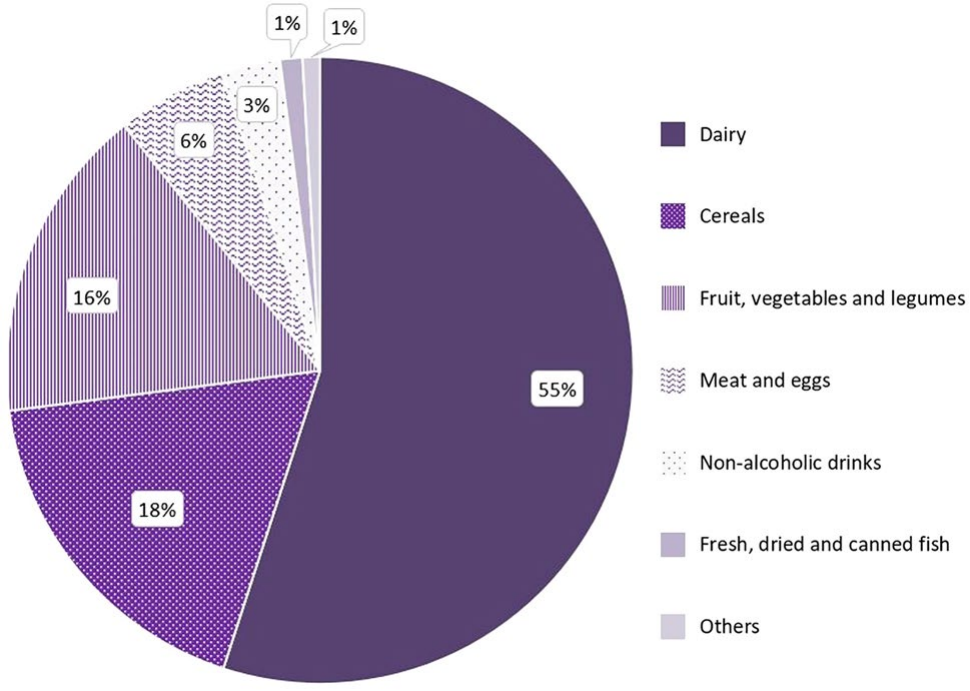
The contribution (%) from food groups to the iMC Salt participant’s total iodine intake by sex

The evaluation of the estimated food intakes and iodine sources showed that dairy, including yoghurt and milk products, was the main source of iodine (Fig. 2), contributing on average with 55% of the iodine intake from diet. Cereals, fruit, vegetables and legumes, and meat and eggs were other food groups with meaningful representation, with contributions of 18%, 16%, and 6%, respectively. No statistically significant difference between sex was observed (*p* > 0.05). The contribution of dairy and cereals was slightly higher in men, whereas the remaining food groups had higher expression in women.

### Estimated iodine intake from household salt

**Fig. 3:**
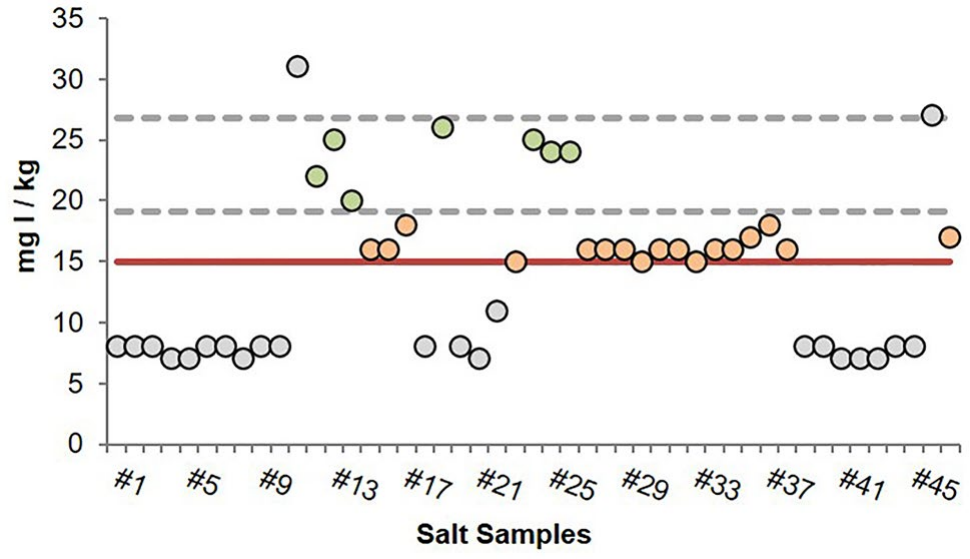
Iodine levels (mg I/kg) found in household salt samples from the iMC Salt intervention group. Green circles—salt samples complying with the WHO and the Portuguese criteria; orange circle—salt samples complying only with the WHO criteria; gray circles—salt samples that did not comply with WHO or the Portuguese criteria; gray dashed line—iodine range according to the Portuguese legal criteria for salt iodization (19–27 mg I/kg); red solid line—WHO-recommended level of iodine for salt iodization (15 mg I/kg)

Integrated into the iMC Salt randomized controlled trial, designed to decrease the salt intake inadequacy, the iodine content in the household salt samples was also analyzed. All the provided 47 salt samples (mostly sea salt) analyzed contained detectable iodine, with an average concentration of 14 mg I/kg (SD 7) (Fig. 3).

Forty-five percent of the samples (21/47) presented iodine concentration below the minimum threshold preconized by WHO (15 mg I/kg). Moreover, only 15% (7/47) of the salt samples showed iodine between the established levels in the non-mandatory iodine fortification Portuguese law (19–27 mg I/kg) [12], with the majority (81%, 38/47) being below the lower limit.

## Discussion

To the best of our knowledge, this is the first study on the iodine status of Portuguese working adults, using gold standard methodology.

Based on previous epidemiological studies performed in school-aged children [5–7, 11], Portugal could be classified as iodine sufficient. However, even though school-aged children are a priority target group and logistically the ideal population to study, they are not representative of the overall population, because differences in diet and metabolism are expected between subpopulations [5]. To what the broad Portuguese population is concerned, no recent data concerning the iodine status are available [5].

With a median 24-h UIC of 66 μg/L, 30% of the population showing 24-h UIC below 50 μg/L, and a daily iodine intake of 112 μg/day, this study revealed that the adult working population had a mild iodine deficiency according to the WHO guidelines for iodine status (median UIC < 100 μg/L plus > 20% of the population with a UIC ≤ 50 μg/L), and the EFSA iodine intake threshold of 150 μg/L. In the literature is argued that the extrapolation from the observed correlation between increasing goiter and a UIE < 100 µg/day to a spot sample UIC below 100 µg/L, as indicative of ID, may have been applicable in children but was not correct for adults [11]. When the urine volume is higher, approximately 1.5 L/day, the UIC in spot samples is usually about 60–65% of the amount excreted in 24 h. In this study, the median 24-h UIE was 94 µg/day, below the threshold value identified in the correlation between UIE and goiter. Unlike other studies, here all the biomarkers and methodologies used to assess the iodine nutritional status agreed on the same classification. Moreover, the median 24-h UIC revealed the same trend reported in adults in other European countries, such as Finland (96 μg/L, [25]), Germany (65 μg/L, [26]), Italy (66 μg/L, [27]), and Norway (88 μg/L, [28]). Indeed, the EUthyroid project corroborated our results, demonstrating that in European countries iodine status is generally adequate in school-aged children, but iodine deficiency may still be present in adults and pregnant women [5]. Also, one possible explanation for the 18% lower iodine intake observed in women in our study may result from higher food consumption by men, including discretionary salt. This iodine intake pattern in what sex is concerned was also noticed in other European studies [27–29].

From a public health perspective, the mild iodine deficiency found in this study reinforces the need to implement supplementation strategies for the whole, but especially for women of childbearing age to meet the increased needs in a future pregnancy and secure a normal development of the fetus. Indeed, iodine inadequacy was found in Portuguese pregnant women more than a decade ago [8], leading to guidelines from the Directorate-General for Health (no. 011/2013), for supplementation in preconception, pregnancy, and breastfeeding. Currently, there is a lack of knowledge concerning the efficiency of the policy implementation. The most recent studies available indicated that the minimum median UIC threshold for iodine adequacy in pregnant women is yet to be achieved [5, 9].

Despite the lower value displayed by the 24-h recall approach, both methodologies applied to estimate the daily iodine intake correlated well. The discrepancy found could be explained by incomplete information on iodine content in foods in the national databases and the self-reported 24-h recall. The 24-h dietary recall indicated that 56.7% of the total iodine intake comes from ingested food. Although the intestinal absorption of ingested iodine is considered to be high (more than 90%), the iodine concentration in water and foods is highly variable [23]. In the present study, the most relevant food sources were dairy, cereals, fruit, and legumes. Milk and dairy are recognized as good sources of iodine, and several studies [e.g., 30–32] reported higher iodine intake in populations with higher consumption levels, including in Portuguese pregnant women [9]. Indeed, iodine content in Portuguese milk and yogurt has been estimated to average 200 and 180 μg/L, respectively [33]. Despite several factors being associated with the iodine concentration in milk, such as farm management, feeding, or seasonality [34, 35], and in accordance with the value found in our study (55%), milk and dairy contribution was reported to be 13–64% of the daily iodine requirement [36].

Cereals, fruit, vegetables, and legumes are generally poor iodine sources, and the levels present depend on the amount of iodine in the soil where the plants are grown. Worldwide, 30% of the population lives in areas with iodine-deficient soil [37], with an average global content of 2.6 mg/kg [38]. The iodine concentration in food crops can be as low as 10 μg/kg [39]. In recent years, to tackle IDD, the biofortification of crops and agricultural soil have been explored [40–44]. The contribution of cereals found in this study can be justified by the consumption of bread and the salt associated with its production. Despite decreasing in recent years, the consumption of bread in the Portuguese population is around 45 kg per year [45], with efforts to reduce the salt content from 10–21 to 11 g/kg in 2021 [46]. Taking the average bread consumption, the aimed value for salt in its production, and the average iodine content in salt found in this study, bread alone can represent a daily iodine intake of nearly 18 µg (12.2% of the daily requirement). Strategies of using iodized salt in bread production have been successfully applied to increase iodine intake adequacy elsewhere [47, 48]. The contribution to the daily iodine intake of fruits, vegetables, and legumes observed in this study is in line with the previously reported iodine content of Portuguese foods [18] and already demonstrated in the literature [49, 50]. An additional problem concerning iodine deficiency is the presence of goitrogens in some popular crops, such as broccoli, cabbage, cauliflower, sweet potato, or soy, with the consequent association of higher consumption to lower iodine status [51, 52].

The contribution of the discretionary salt to the daily iodine intake was 43% (52 μg), estimated by the difference between the total daily intake (mean 121 μg/day based on UIE), and the iodine amount provided by food (mean 69 μg/L based on the 24-h dietary recall). In the IMC Salt study, the average salt intake at baseline was 8 g/day [15], of which about 3.3 g per day was discretionary salt (41% [53]). Taking into account the average iodine concentration found in the studied household salt (14 mg I/kg), the discretionary salt accounted for 47 µg of the daily iodine intake (38%). To note that, when the contribution of discretionary salt is added to the contribution of ingested food based on the 24-h dietary recall, the gap between approaches applied is minimum (121 μg/day based on UIE, and 116 μg/day based on the 24-h dietary recall plus discretionary salt contribution). Thus, the discretionary salt contribution and the daily iodine intake determined by the two data sets collected in this study seem to agree well, conferring reliability and robustness to the results. Also, the mean iodine content in household salt was similar to the reported in a previous study concerning commercially available salt in the same area (13.8 mg I/kg, [20]. Moreover, this percentage of discretionary salt contribution to the daily iodine intake is in line with the values reported for other studies in European countries, such as Italy and Germany [54, 55].

Considering the sodium-healthy diet, as preconized by WHO [56], which recommends the consumption of up to 5 g of salt per day, the iodine intake from salt would be around 56 µg/day, 23 µg of which was from discretionary salt. Taking the contribution of food estimated in this study, the daily iodine intake would be 92 µg, far from the recommended 150 µg. Even taking the estimated 11 g/day average salt intake by Portuguese adults described in the literature [57], retaining the highest percentage of discretionary salt use reported (25–50%, [19, 58]), and assuming no loss during cooking, the iodine intake (144 µg/day) would not be sufficient to meet the guidelines.

The iodization of the salt used in households is the primary strategy to prevent iodine deficiency at a population level [1]. In Portugal, most salt used at the household level and in the food industry is of marine origin (sea salt). In the country, the iodine fortification of salt is not mandatory, with the consumption being limited, in the range of 2–8.8% [6, 59], owing to the higher sales price compared to non-iodized sea salt. However, the non-mandatory iodine fortification Portuguese law established a salt fortification range between 19 and 27 mg I/kg in the iodide form [12].

If an iodoprophylaxis was followed, with the fortification of the household and food industry salt, and retaining the average contribution of iodine from food estimated in this study, the daily iodine intake would be 168–199 µg in a sodium-healthy diet, already accounting for the inevitable 20% lost during cooking [1]. In this scenario, the population would be iodine sufficient according to the WHO and EFSA threshold for iodine intake. Moreover, since the mean concentration of iodine in Portuguese marine salt is already close to the WHO-recommended iodine threshold of 15 mg I/kg [60], the associated production costs of fortification would be smaller.

### Study strengths and limitations

The iodine intake was estimated simultaneously from both the 24-h urinary iodine excretion and a detailed 24-h dietary recall for each participant. Although cumbersome, the 24-h urinary iodine excretion is considered the best biomarker to assess and monitor the recent iodine intake of a given population [61]. Also, the concurrent creatinine content analysis allowed the exclusion of incomplete urine collections, making the results more robust. The use of the 24-h dietary recall enabled the estimation of the amount of iodine derived from food as well as the indirect contribution of discretionary salt. Moreover, the iodine direct quantification in household salt was also important to understand the current role of salt in iodine intake and the potential impact of the consumption reduction. Additionally, since most iodine comes from food, the comparison between methodologies allowed us to understand if the approaches were interchangeable. A major strength of this study was to demonstrate the potential of the mandatory occupational health appointments in the education and monitoring of the iodine subject, increasing the quality of life, and consequent productivity of the working population and associated households.

A major limitation of the study was the relatively small sample size. The data collection was performed during the COVID-19 pandemic, and despite the efforts made to solve the inherent implications, it was impossible to achieve the sample size initially estimated [16]. Moreover, the study population was not nationally representative, since participants were primarily recruited among the university staff, mainly from coastal urban areas, with a high level of education, and included fewer smokers than the general population. Regional divergence, with proximity to the coastal zone and rural areas associated with the iodine status, has been reported, including in Portugal [6, 7, 62, 63]. Also, participants with a higher level of education were more likely to have better socio-economic status and therefore be more prone to a healthy diet. Although being a controversial topic, several studies linked IDD with lower socio-economic status and the associated access to iodine-rich foods [e.g., 64–66]. Another limitation can be the use of a single 24-h urine collection, since it is known that several collections are required to reduce the day-to-day variation and estimate the iodine intake accurately [67]. Also, the 24-h dietary recall data are self-reported, and the daily iodine intake estimation relies on iodine content in foods found in traditionally incomplete national databases, which can lead to under- or overestimation of iodine intake. Finally, the lack of direct information about the amount of discretionary salt or the use of iodized salt in the household of the participants limited the understanding of the salt contribution to the daily iodine intake.

## Conclusions

To the best of our knowledge, this was the first study reporting on the iodine status in Portuguese working adults, using gold standard methodology. The results revealed a moderate iodine deficiency, with higher expression in women. Dairy was described as the major iodine source in the diet. Although the discretionary salt accounted for almost half of the iodine intake, the unfortified household marine salt failed to provide the required daily intake. Taking into consideration the present iodine status of the country, and the WHO major priority in salt reduction to tackle non-communicable diseases, it is pivotal to design public health strategies and monitoring programs to ensure iodine adequacy in all population groups. In this setting, the mandatory occupational health appointments that this study took advantage of have proven to be an asset for future monitoring programs and in education toward the use of iodized salt.

## Data Availability

The datasets used and/or analyzed during the current study are available from the corresponding author on reasonable request.
